# An evaluation of venous thromboembolism by whole-body enhanced CT scan for critical COVID-19 pneumonia with markedly rises of coagulopathy related factors: a case series study

**DOI:** 10.1186/s12959-021-00280-z

**Published:** 2021-04-20

**Authors:** Fumihiro Ogawa, Yasufumi Oi, Kento Nakajima, Reo Matsumura, Tomoki Nakagawa, Takao Miyagawa, Takeru Abe, Ichiro Takeuchi

**Affiliations:** Department of Emergency Medicine, Yokohama City University, School of Medicine, 3-9 Fukuura, Kanazawa-ku, Yokohama, Kanagawa 236-0004 Japan

**Keywords:** COVID-19, Venous thromboembolism, Whole-body CT scan, D-dimer, Coagulopathy, Anticoagulant

## Abstract

**Background:**

Coronavirus disease (COVID-19) pneumonitis associated with severe respiratory failure has a high mortality rate. Based on recent reports, the most severely ill patients present with coagulopathy, and disseminated intravascular coagulation (DIC)-like massive intravascular clot formation is frequently observed. Coagulopathy has emerged as a significant contributor to thrombotic complications. Although recommendations have been made for anticoagulant use for COVID-19, no guidelines have been specified.

**Case summary:**

We describe four cases of critical COVID-19 with thrombosis detected by enhanced CT scan. The CT findings of all cases demonstrated typical findings of COVID-19 and pulmonary embolism or deep venous thrombus without critical exacerbation. Two patients died of respiratory failure due to COVID-19.

**Discussion:**

Previous reports have suggested coagulopathy with thrombotic signs as the main pathological feature of COVID-19, but no previous reports have focused on coagulopathy evaluated by whole-body enhanced CT scan. Changes in hemostatic biomarkers, represented by an increase in D-dimer and fibrin/fibrinogen degradation products, indicated that the essence of coagulopathy was massive fibrin formation. Although there were no clinical symptoms related to their prognosis, critical COVID-19-induced systemic thrombus formation was observed.

**Conclusions:**

Therapeutic dose anticoagulants should be considered for critical COVID-19 because of induced coagulopathy, and aggressive follow-up by whole body enhanced CT scan for systemic venous thromboembolism (VTE) is necessary.

**Supplementary Information:**

The online version contains supplementary material available at 10.1186/s12959-021-00280-z.

## Background

The coronavirus disease (COVID-19) pandemic, as declared by the World Health Organization, is caused by the severe acute respiratory syndrome coronavirus 2 (SARS-CoV-2) [1, 2]. Recent studies have reported a high prevalence of thrombotic events in COVID-19 [3, 4]. In particular, venous thromboembolism (VTE) has emerged as an important consideration in the management of hospitalized COVID-19 patients. Early reports suggested a high incidence of VTE in hospitalized COVID-19 patients, particularly those with severe illness. This was similar to the high VTE rates observed in patients with other viral pneumonias, including severe acute respiratory syndrome (SARS) and Middle East respiratory syndrome (MERS-CoV) [5–8]. COVID-19 is associated with abnormalities in markers of hypercoagulability, including elevated levels of D-dimer, fibrin/fibrinogen degradation products (FDP), fibrinogen, and factor VIII, shortened activated partial thromboplastin time (APTT), and elevated sepsis-induced coagulopathy (SIC) score [9]. Hospitalized COVID-19 patients share similar strong clinical intrinsic and extrinsic risk factors for VTE, which include advanced age, obesity, immobility/stroke with paralysis, a history of cancer/active cancer, management in an intensive care unit setting, and prior history of VTE or known thrombophilia [9, 10]. However, risk stratification for VTE and the optimal intensity and duration of anticoagulant thromboprophylaxis, including post-hospital discharge prophylaxis, remain uncertain in hospitalized COVID-19 patients.

The severity classification and treatment strategies used for patients with critical COVID-19 in our institute is shown in Table 1. Additionally, for COVID-19 respiratory distress, we strictly decide to introduce ECMO based on the ELSO criteria [11]. Upon admission to our hospital, we diagnosed COVID-19 based on a positive reverse transcriptase–polymerase chain reaction (RT-PCR) assay for SARS-CoV-2 using nasopharyngeal and laryngeal swab samples that were analyzed in a designated diagnostic laboratory.

**Table 1:** Severity Classification Criteria and Therapeutic Strategy for critical COVID-19

● Criteria for severe COVID-19		
1) SpO_2_ < 92% at 10 L/min. Oxygen via a reservoir mask		
2) Shortness of breath with respiratory rate of > 30/min.		
3) Severe dyspnea due to COVID-19 pneumonia		
● Therapeutic strategy for critical COVID-19 [11]		
1) Mechanical ventilator	mode	pressure control
(primary setting)	PEEP	10–15 mmH_2_O
	Driving Pressure	20–25 mmH_2_O
	Respiratory Rate	12–16/min.
2) Antiviral therapy	Remdesivir [12]	10 days
3) Systemic steroid therapy	Dexamethasone [13]	10 days
4) Anticoagulant therapy	UFH with therapeutic dose according to APTT (1.5–2 times as normal)	
5) Protection for DVT	Intermittent air compression and elastic stocking	
6) Antibiotics	for CAP or secondary bacterial or fungus infection	
7) Rehabilitation	early intervention by NS, PT and OT	
8) Nutrition	early intervention via tube feeding or TPN	
9) Supportive therapy	sedation,catecholamine support etc. via central venous catheter	

*PEEP* Positive end-expiratory pressure, *PS* Pressure support, *UFH* Unfractionated heparin, *APTT* Activated partial thromboplastin time, *CAP* Community associated pneumonia, *NS* Nurse, *PT* Physical therapist, *OT* Occupational therapist, *TPN* Total parenteral nutrition

Here, we describe four cases of critical COVID-19 with venous thrombosis that required admission to the intensive care unit.

## Cases

We have treated 42 critical COVID-19 patients with intubation in our intensive care unit, since February 2020, and have performed enhanced computed tomography (CT) scans for 15 critical COVID-19 patients after extubation or during mechanical ventilation to follow up their lung injury status. We obtained written informed consent from all patients prior to their intubation. Of these, 11 patients did not have VTE and four patients had critical COVID-19 with VTE as shown by the chest CT findings, as shown in Table 2. The characteristics of the patients with VTE did not differ significantly from those of the other patients.

**Table 2:** Patients’ Characteristics

	non-VTE (*n* = 11)	VTE (*n* = 4)	*p* value*
Age (year-old; median [IQR])	72 [68–78]	65 [63–69]	0.4255
Gender (Male: %)	91	75	0.4762
BMI (median [IQR])	23.7 [23–25]	23.5 [23–25]	0.8939
First Symptom (%)			
dyspnea	36	25	1.0000
fever	91	100	1.0000
cough	18	25	1.0000
Admission day after onset (day; median [IQR])	10 [8–11]	9 [8–11]	0.9272
Smoking History (%)	82	75	1.0000
Comorbidity (%)			
Diabetes	64	0	0.0769
Renal Dysfunction	36	50	1.0000
Hemodialysis	36	0	0.5165
Hypertension	82	50	0.5165
Hyperlipidemia	64	100	0.5165
Hyperuricemia	45	25	0.6044
Cardiovascular Disease	9	0	0.4762
Respiratory Disease	9	0	1.0000
Cancer	18	25	1.0000
Collagen Disease	0	25	0.2667
Thrombotic disease	36	0	0.5165
others	18	50	0.5165

*Mann-Whitney U test or Fisher’s exact test; *IQR* Interquartile range, *BMI* Body Mass Index

The patients with VTE (Table 3), were aged 59–79 years and had symptoms of only high fever and cough. Three of the patients were smokers. The patients comorbidities were as follows: two had renal dysfunction without hemodialysis and hypertension, all patients had hyperlipidemia, and one was on oral systemic steroid therapy for systemic lupus erythematosus (SLE). None of the patients had diabetes mellitus. The average number of days of illness before admission to our hospital and the start of our therapeutic strategy was 9.5 days (range: 6–14 days) after the onset. As a therapeutic strategy to prevent VTE, we maintained the APTT value in the range of 1.5–2 times the control value with continuous intravenous unfractionated heparin (UFH) at a therapeutic anticoagulant dose during intubation and UFH 5000 U twice a day by subcutaneous injection after extubation in all critical COVID-19 patients. Recently, we have been performing systemic enhanced CT the day after extubation as a routine examination. Earlier, we performed additional systemic enhanced CT scan only when we suspected VTE based on the physical findings or the laboratory thrombotic indicator data, such as elevated D-dimer and FDP. And, we routinely performed four-points thrombus check by sonography to prevent from thromboembolism.

**Table 3:** Detailed characteristics of VTE patients

Patient	1	2	3	4
Age	66	59	79	64
Gender	Male	Male	Female	Male
BMI	28.0	22.6	22.4	24.4
First Symptom	fever	fever	cough	fever
Admission day after onset	8	14	10	6
Smoking History	+	+	–	+
Comorbidity				
Diabetes	–	–	–	–
Renal Dysfunction	+	+	–	–
Hemodialysis	–	–	–	–
Hypertension	+	+	–	–
Hyperlipidemia	+	+	+	+
Hyperuricemia	+	–	–	–
Cardiovascular Disease	–	–	–	–
Respiratory Disease	–	–	–	–
Cancer	–	–	+; Ov	–
Collagen Disease	–	+; SLE	–	–
others	–	+; HBV	–	+; HBV

*BMI* Body Mass Index, *Ov* Ovarian Cancer, *SLE* Systemic Lupus Erythematosus, *HBV* Hepatitis B virus

### Case 1

**Fig. 1:**
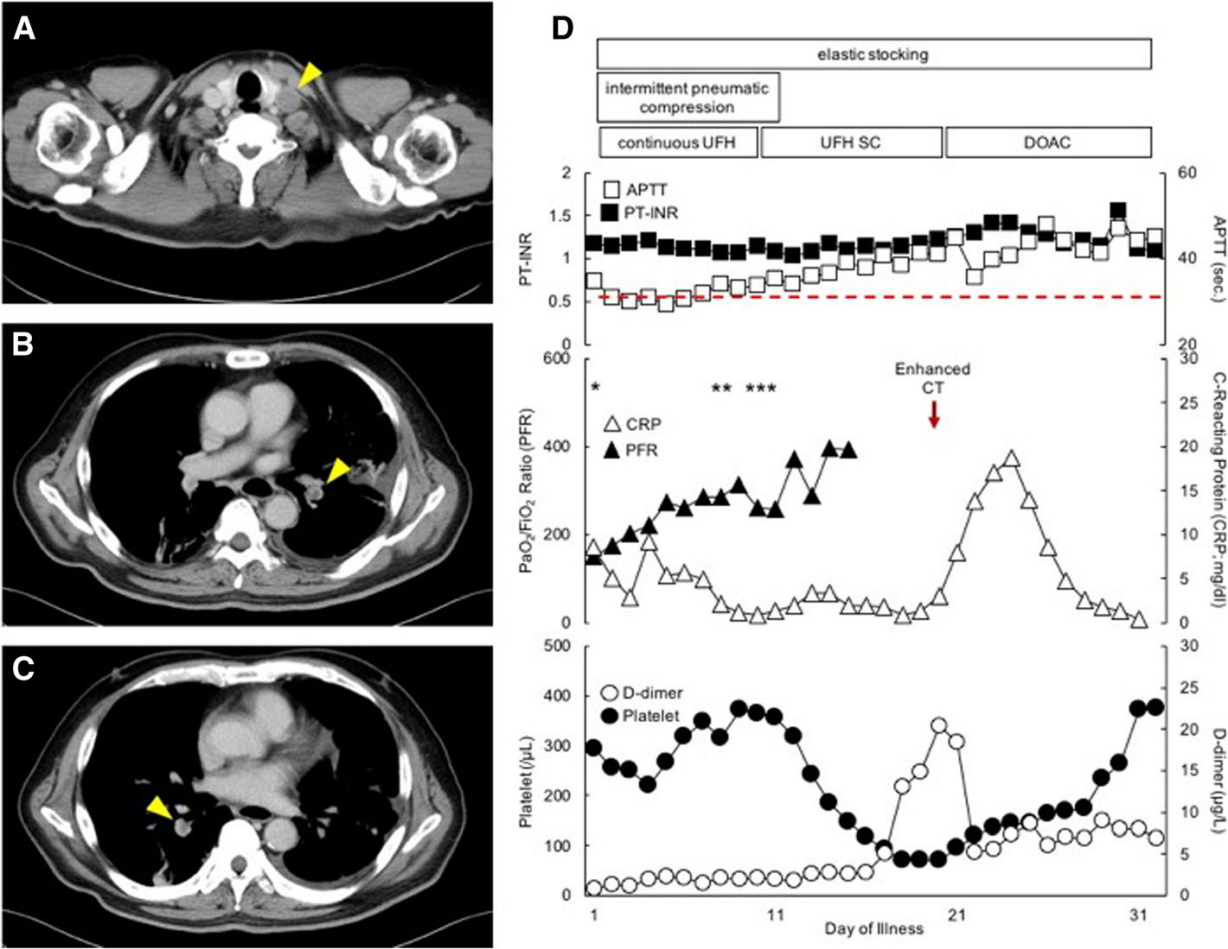
Computed tomography (CT) scan images and time course of PFR, CRP, D-dimer and platelet in case 1: (**a**, **b**, **c**) enhanced CT images (arrow head indicated thrombus); (**d**) clinical course. X-axis: day of illness (day). Y-axis: each parameter. Abbreviations: PT-INR: prothrombin time-international normalized ratio; APTT: activated partial thromboplastin time; PFR: PaO_2_/FiO_2_ ratio; CRP: C-reacting protein; UFH: unfractionated heparin; SC: subcutaneous injection; DOAC: direct oral anticoagulant. Red border line; standard value of APTT. *, intubation, **, extubation, ***, discharge from intensive care unit

A 66-year-old man diagnosed with critical COVID-19 and fever was transferred to our hospital. He had renal dysfunction (without hemodialysis), hypertension, hyperlipidemia, and hyperuricemia. After intubation, his condition gradually improved with our therapeutic strategy and he was extubated on the 8th day of admission. After he was moved to the general ward for rehabilitation, a sudden increase in D-dimer (20.47 μg/mL) was observed with a concurrent increase in FDP (Supplement Fig. 1). We suspected VTE, which prompted enhanced CT screening. Internal jugular vein thrombosis (Fig. 1a) at the site of the central venous catheter, and pulmonary embolism (PE) (Fig. 1b, c) on the proximal side of the bilateral pulmonary artery were detected. We used direct oral anticoagulants as the standard treatment for PE, based on the advice of a cardiologist. At this time, the patient had no symptoms or signs such as dyspnea, tachypnea, or chest pain. His rehabilitation was completed, and no conspicuous sequelae of COVID-19 were observed, and the patient was discharged from the hospital on the 35th day of admission. The patient’ s clinical course is shown in Fig. 1d. One month after discharge, we could not detect PE on follow-up enhanced chest CT.

### Case 2

**Fig. 2:**
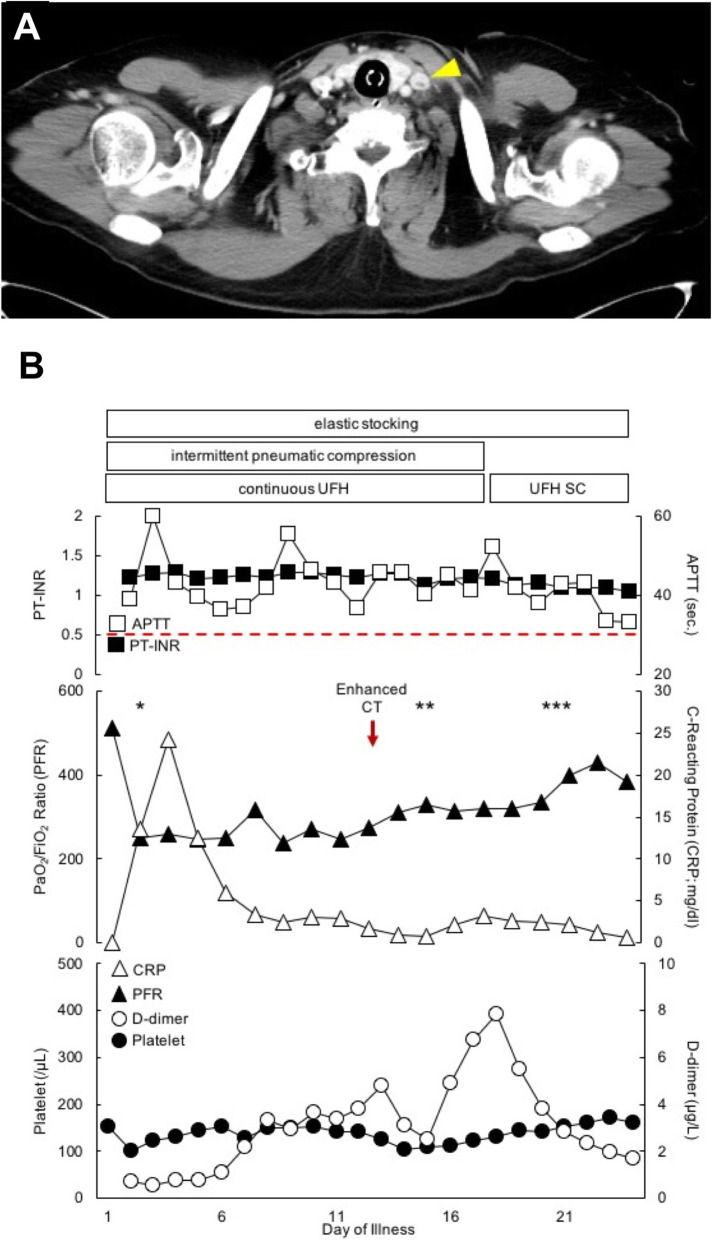
CT scan images and time course of PFR, CRP, D-dimer and platelet in case 2: (**a**) enhanced CT image (arrow head indicated thrombus); (**b**) clinical course. X-axis: day of illness (day). Y-axis: each parameter. Abbreviations: PT-INR: prothrombin time-international normalized ratio; APTT: activated partial thromboplastin time; PFR: PaO_2_/FiO_2_ ratio; CRP: C-reacting protein; UFH: unfractionated heparin; SC: subcutaneous injection; Red border line; standard value of APTT. *, intubation, **, extubation, ***, discharge from intensive care unit

A 59-year-old man diagnosed with critical COVID-19 with fever was admitted to our hospital. He was on oral prednisolone for SLE and had hypertension, hyperlipidemia, and hepatitis B virus infection for which he was on oral entecavir hydrate. After intubation, his condition gradually improved. His D-dimer level (7.83 μg/mL) gradually increased without any symptoms, with a parallel increase in the FDP level and an inversely proportional decrease in the fibrinogen level that were measured concurrently (Supplement Fig. S2), which necessitated enhanced CT screening. An internal jugular vein thrombosis (Fig. 2a) was found at the site of the central venous catheter, without other signs of VTE. The therapeutic dose of UFH was used as the standard treatment for internal jugular vein thrombosis. His condition improved, his requirement for ventilatory support decreased, and the inflammatory response also steadily reduced, so the patient was extubated on the 15th day of admission. After extubation, repeated the whole-body CT scan., We could not detect any residual internal jugular vein thrombus, so, we changed the patient from continuous UFH to subcutaneous injections of UFH. His systemic condition was stable, and he completed rehabilitation, and was followed up for SLE in the Department of Collagen Disease. He was discharged from the hospital on the 26th day of admission. The patient’ s clinical course is shown in Fig. 2b.

### Case 3

**Fig. 3:**
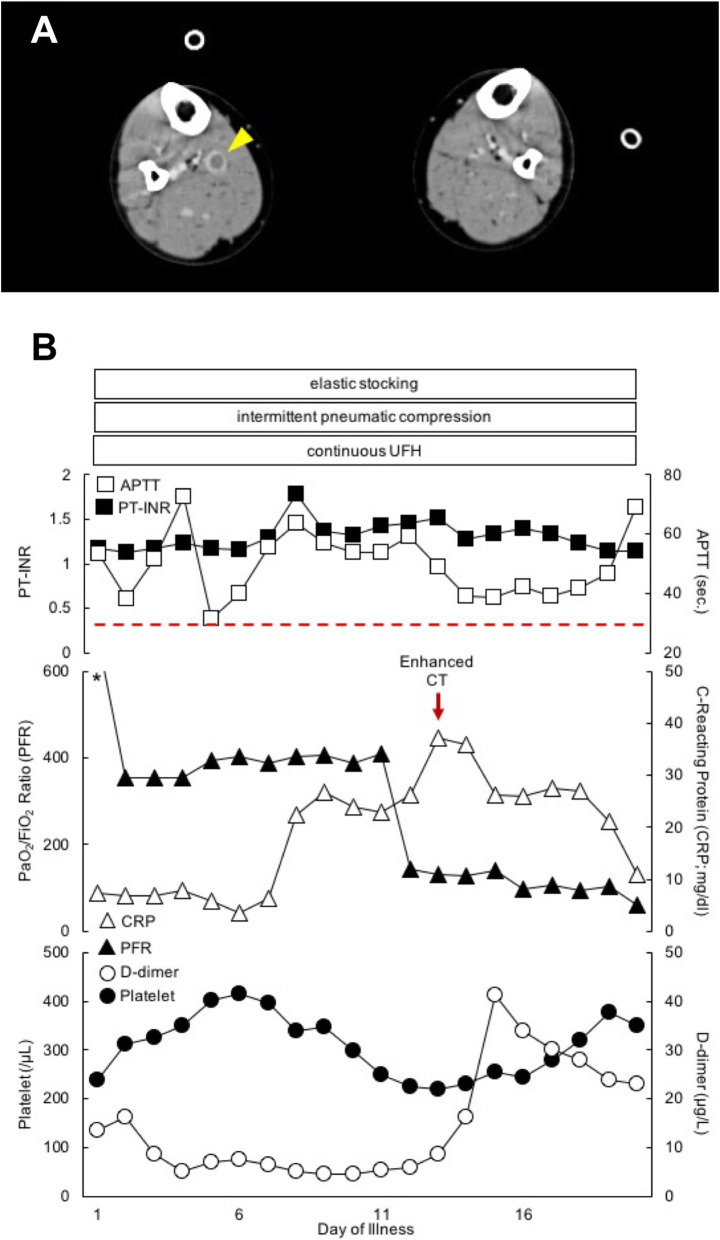
CT scan images and time course of PFR, CRP, D-dimer and platelet in case 3: (**a**) enhanced CT image (arrow head indicated thrombus); (**b**) clinical course. X-axis: day of illness (day). Y-axis: each parameter. Abbreviations: PT-INR: prothrombin time-international normalized ratio; APTT: activated partial thromboplastin time; PFR: PaO_2_/FiO_2_ ratio; CRP: C-reacting protein; UFH: unfractionated heparin; Red border line; standard value of APTT; *, intubation

A 79-year-old woman diagnosed with critical COVID-19 with cough was admitted to our hospital. She had hyperlipidemia and cured ovarian cancer as. After intubation, her condition worsened, without any evidence of bacterial infection. During mechanical ventilation, the D-dimer level suddenly increased to 8.58 μg/mL with a concurrent increase in the FDP (Supplement Fig. S3), which prompted enhanced CT screening. As a result, a deep vein thrombosis (DVT) was found in the right popliteal vein without other VTEs (Fig. 3a). We used therapeutic-dose UFH as the standard treatment for DVT. Her general condition and inflammatory response worsened, and her oxygenation decreased, which subsequently led to her death on the 21st day of admission. We thought that the cause of death in this case was not VTE, but directly due to COVID-19 itself. The patient’ s clinical course is shown in Fig. 3b.

### Case 4

**Fig. 4:**
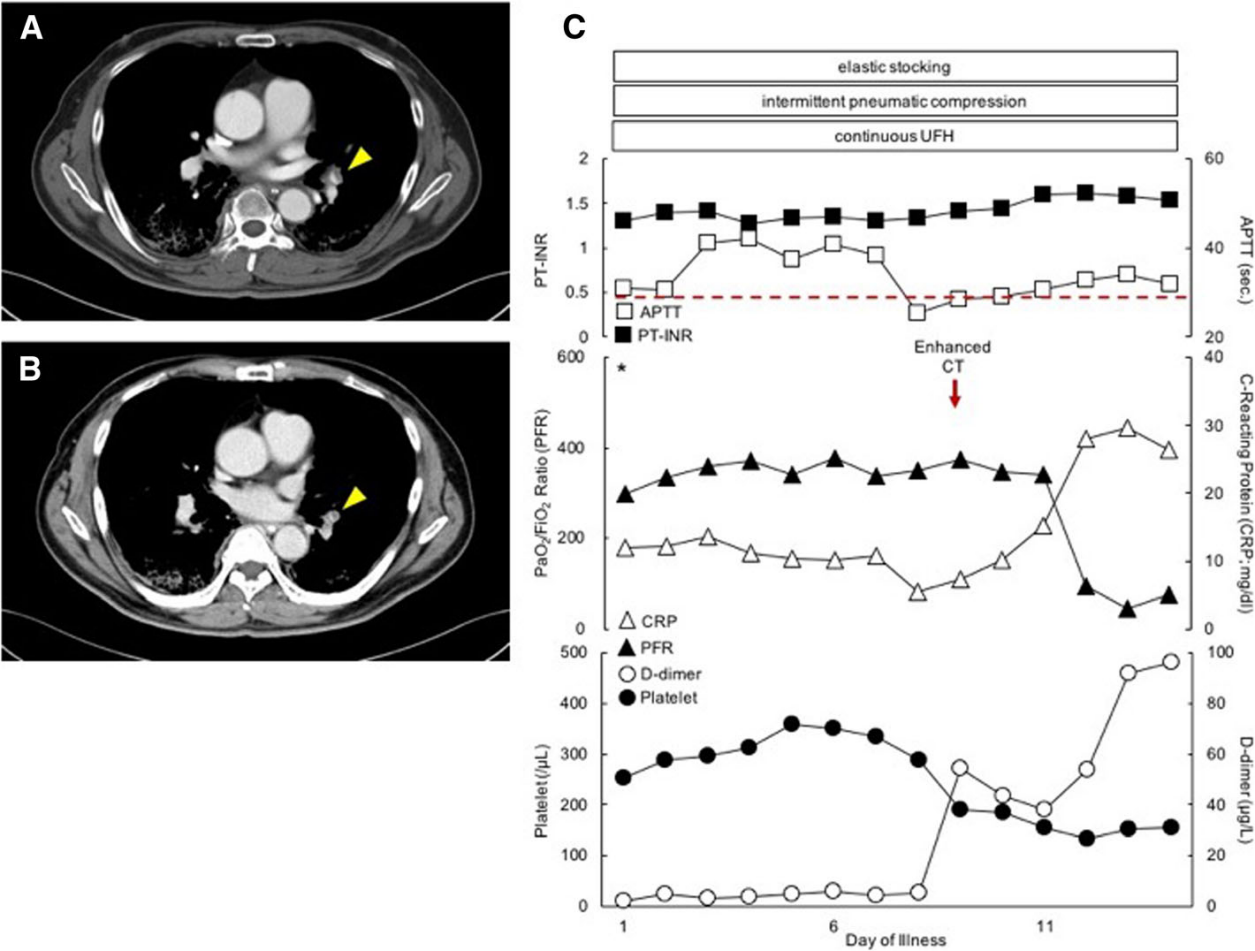
CT scan images and time course of PFR, CRP, D-dimer and platelet in case 4: (**a**), **b** enhanced CT images (arrow head indicated thrombus); **c** Clinical course. X-axis: day of illness (day). Y-axis: each parameter. Abbreviations: PT-INR: prothrombin time-international normalized ratio; APTT: activated partial thromboplastin time; PFR: PaO_2_/FiO_2_ ratio; CRP: C-reacting protein; UFH: unfractionated heparin; Red border line; standard value of APTT *, intubation

A 64-year-old man diagnosed with critical COVID-19 and fever was transferred to our hospital from another hospital. He had hyperlipidemia and hepatitis B virus infection, which was being treated with oral entecavir hydrate, as comorbidities. After intubation, his condition gradually improved. However, while on mechanical ventilation, his D-dimer level suddenly increased (54.53 μg/mL) with a concurrent increase in the FDP level and decrease in fibrinogen level that were measured at the same time (Supplement Fig. S4), and so we suspected VTE, which prompted enhanced CT screening. As a result, a massive PE was detected on the distal side of the left pulmonary artery (Fig. 4a, b). We continued to use therapeutic-dose UFH as the standard treatment for PE. However, his condition suddenly worsened on the 12th day of admission, and he died on the 14th day of admission. We were unable to determine whether his cause of death was massive PE or deterioration due to COVID-19 itself. The patient’s clinical course is shown in Fig. 4c.

In all cases, the International Society on Thrombosis and Hemostasis (ISTH) score in the VTE group was less than 5 pts., the SIC score was less than 4 pts., and there was no evidence of disseminated intravascular coagulation (DIC) in their clinical course. After these results, we performed plain chest CT scan after extubation as a follow up of the lung condition in critical COVID-19 patients. However, we routinely performed whole-body enhanced CT in all cases to follow up the condition of their lungs and other organs for the origin of infection, and to search for whole-body VTE if there was a rapid increase in the D-dimer level or an originally high D-dimer in a critical COVID-19 patient.

## Discussion

There were no dramatic differences in their clinical course of the VTE patients and non-VTE patients, except for the rapid elevation of D-dimer levels in the patients with VTE. All patients were provided with fundamental therapy for COVID-19, rehabilitation, nutrition, and supportive therapy. Patients 1 and 2 did not have massive VTE; in particular, Patient 1 had a thrombus after extubation during rehabilitation in the intensive care unit, but his respiratory condition, blood pressure, and other vital signs were almost stable without any symptoms. In contrast, Patients 3 and 4 had massive VTE, but we were unable to establish the cause of their death. Patient 3 had VTE of only one popliteal vein without lethal PE, so the reason for her death was not VTE, but COVID-19 itself. Case 4 may have died from massive PE associated with COVID-19 because he experienced a dramatic clinical decline after his D-dimer level became elevated and his blood oxygen became desaturated due to PE. The exact timing of thrombosis in the course of the disease, and the optimal treatment for COVID-19 thrombosis remain unknown, but our experience suggests that comprehensive imaging should be considered soon after the presentation, as thrombosis may occur early and this warrants therapeutic-dose anticoagulant therapy. We inserted a central venous catheter for severe COVID-19 patients. Catheters tend to form a thrombus in the jugular vein or other insert point, as in the two patients with jugular vein thrombus. Clearly, any benefit of thrombolysis should be balanced against the risk of bleeding, which was often considerable in ICU patients and can lead to intracranial hemorrhage [12]. Compared with white individuals of European descent, the incidence is higher in black individuals of African descent and lower in Asian individuals [13], a disparity for which the reason is unknown, but there is a high level of concern regarding the risk of bleeding complications in Asian countries, which leads to the general reluctance for to use anticoagulants to prevent VTE. There is little information available on the risk of hemorrhage in Asian patients [14]. Two cases of hemorrhagic complications occurred our COVID-19 cohort, in the non-VTE group, and the patients developed retroperitoneal hemorrhage and intra-abdominal hemorrhage. Both patients had severe diabetes mellitus, chronic renal failure, and hemodialysis as comorbidities, and one patient had been on long-term steroids for lung lesions, complicated by thrombocythemia caused by COVID-19. It has been reported that COVID-19 may cause DIC of the fibrinolytic type from sepsis caused by viral infection, [15], but only one of our case patients, who had a bleeding tendency, developed DIC. Therefore, it is important to pay attention to the possibility of hemorrhagic complications in high-risk patients.

Critical COVID-19 patients displayed coagulation abnormalities associated with respiratory deterioration and death [16, 17]. In addition, many critical COVID-19 patients developed venous thromboembolism, which appeared to be related to coagulopathy [7, 18]. In particular, VTE emerged as an important consideration in the management of hospitalized patients with COVID-19. However, these observations may have been limited by the low rates of cross-sectional imaging performed (10%) as reported in one study [19]. In recent years, common pathways for venous thrombosis have been described, with inflammation and hypercoagulation being key factors in the mechanism of venous thrombotic events [20]. These concerns should be balanced by emerging data that the incidence of VTE in hospitalized critical COVID-19 patients or in ICU settings was higher than that reported by historical data in similar patients, with an incidence of VTE of 27% in a previous study using standard thromboprophylaxis and an incidence of 25% in another study without prophylaxis [5, 7]. These findings were consistent with high rates of VTE in patients with other severe viral pneumonias, such as influenza H1N1, in whom there was an 18- to 23-fold higher risk for VTE compared with control patients [8]. Although the mechanisms underlying vascular thrombosis in COVID-19 have not yet been clearly defined, several have been postulated. The tropism of the virus for the angiotensin-converting enzyme-2 (ACE2) receptor of the endothelial cells resulted in endotheliopathy and endothelial cell apoptosis [21]. Activation of the complement system led to endothelial cell injury and death with subsequent vascular denudation and exposure of the thrombogenic basement membrane, which drives the activation of clotting cascades. These events resulted in inflammation, microvascular thrombosis, vessel edema, and hemorrhagic sequelae, all of which were prominent features of lung pathology in patients with COVID-19-associated pneumonia. The pulmonary thrombosis in severe COVID-19 can be described as macroscopic and microscopic thrombosis. Although activation of coagulation is linked to a systemic cytokine storm, as the principal site of thrombus formation is the lungs, it might also be referred to as pulmonary intravascular coagulation [22]. In an autopsy study of ten patients with COVID-19, small vessel thrombus formation in the lung periphery was associated with foci of alveolar hemorrhage [23]. Additionally, previous reports have noted that fibrin micro-thrombus that was frequently seen at the level of the pulmonary microcirculation in autopsy investigations without detection of VTE by enhanced CT scan [23–25]. Therefore, it is necessary to perform anticoagulant therapy based on the assumption of the presence of microthrombosis that can’t be detected by diagnostic imaging such as systemic enhanced CT scan. Two of our patients with VTE died after the VTE was detected, but we do not know their final cause of death. VTE may be a prognostic factor for critical COVID-19 patients.

The diagnostic assessment of suspected VTE in hospitalized COVID-19 patients is challenging, especially for critically ill patients in whom, typically, it is important to reliably confirm or exclude VTE. Imaging studies for DVT or PE may be avoided due to concerns about transmitting infection in non-COVID-19 hospital wards or to healthcare workers. The frequent finding of an elevated D-dimer level in severely hospitalized COVID-19 patients may prompt an aggressive diagnostic approach for VTE, despite the controversy that an elevated D-dimer level (> 4.0 mg/L) may not be a reliable predictor of VTE in this population, but rather a marker of poor overall outcome [5, 26]. A recent study found an 85.0% sensitivity and an 88.5% specificity for diagnosing VTE in patients with D-dimer levels > 1.5 mg/L [5].

In our critical COVID-19 patients, almost all patients had elevated D-dimer levels (> 4.0 mg/L), and except for six critical COVID-19 patients, we could not detect VTE by systemic enhanced CT scan. As one of the natural courses of coagulopathy, D-dimer levels on the day of admission were mostly elevated to levels > 4.0 mg/L, peaked a few days later, decreased during the recovery period of the disease, and normalized gradually. In a previous study, D-dimer and FDP were observed to increase in parallel, which suggested a common coagulation activation and secondary hyperfibrinolysis condition. Markedly elevated D-dimer and FDP levels are prognostic factor for severe COVID-19 patients [16]. In these four patients, their coagulation data showed rapid elevation of D-dimer with the rapid elevation of FDP, and decreased platelet count before the VTEs were detected by enhanced CT scan. In our study, there were two deaths amount patients with an extremely rapid elevation of D-dimer and FDP levels and VTE shortly before death. Therefore, the rapid change of elevated D-dimer and decreased platelet count may be an index to check VTE by enhanced CT scan during the clinical course of critical COVID-19.

The World Health Organization recommends therapeutic anticoagulation rather than intermediate dosing [27], but the optimal thromboprophylaxis strategy in the critically ill hospitalized COVID-19 patients is uncertain (conditional recommendation, very low certainty). A previous report suggested that the use of either prophylactic or intermediate doses of low molecular weight heparin (LMWH) in critical COVID-19 was associated with improved outcomes and better prognosis [28]. A previous report that assessed a therapeutic-dose of UFH in patients with acute respiratory distress syndrome who were afflicted with influenza virus, found that patients with acute respiratory distress syndrome who received therapeutic-dose anticoagulation had 33-fold fewer VTE events than those treated with prophylactic UFH or LMWH [8]. In addition to intensive care management, thrombotic tendencies in COVID-19 promoted VTE formation, so therapeutic anticoagulant doses were more appropriate than intermediate doses. Since LMWH had no control index, we preferred to use UFH, which can be monitored by the APTT value while paying attention to side effects such as bleeding. Nevertheless, during anticoagulant therapy with UFH we tried to maintain an APTT value at 1.5–2 times the control value, to prevent VTE. But some cases were uncontrollable even if the UFH was over than 20,000 U/day continuous UFH. Four patients developed uncontrollable VTE. Therefore, as coagulation ability varies from person-to-person, it was necessary to check other coagulation-related factors. The use of empiric therapeutic-dose anticoagulation has been advocated by some for critically-ill and hospitalized COVID-19 patients, especially in ICU settings; however, data on the efficacy and safety of this approach are limited [7]; We must prevent VTE by rigorous multimodal prophylaxis strategies (anticoagulant and mechanical) in the critically ill and completely immobile COVID-19 population [9]. We need further results of trials to assess the efficacy and safety of dose of anticoagulant in hospitalized COVID-19 patients.

This study had some limitations. First, this study was performed in a single hospital with a small study population, as there are currently few confirmed and recovered cases of COVID-19 in Japan. Second, no therapeutic treatment for VTE in patients with severe COVID-19 was available for use in a parallel control group. However, we believe that the credibility of the therapeutic effect is high, as our study provides a comprehensive examination, including clinical features, laboratory findings, and physical findings, at a single institution. Third, we did not check for antithrombin III (ATIII), factor Xa, protein S, and protein C. Previous reports suggest that the effects and complications of heparin show individual differences between metabolism and some enzymes [14, 29]. Therefore, these factors should be checked in COVID-19 patients during intensive care. In the future, we hope to collaborate with other medical institutions in our area to design a control group that will allow us to improve the reliability of our study.

## Conclusion

It is very important for critical COVID-19 patients’ therapeutic-dose anticoagulants to be strictly monitored by APTT tests, and for whole-body enhanced CT to be performed for the detection of VTE and the follow-up of these patients. Further studies on detailed and early laboratory, clinical, and imaging characterization are needed to better understand the pathophysiology of the thrombotic nature of COVID-19.

## Supplementary Information

**Additional file 1: Supplement Figure S1.** Time course of d-dimer and FDP level in Case 1. X-axis: day of illness (day). Y-axis: each parameter. Abbreviations: FDP: fibrin/fibrinogen degradation products; *, intubation, **, extubation, ***, discharge from intensive care unit.

**Additional file 2: Supplement Figure S2.** Time course of d-dimer and FDP, d-dimer and fibrinogen level in Case 2. X-axis: day of illness (day). Y-axis: each parameter. Abbreviations: FDP: fibrin/fibrinogen degradation products; *, intubation, **, extubation, ***, discharge from intensive care unit.

**Additional file 3: Supplement Figure S3.** Time course of d-dimer and FDP level in Case 3. X-axis: day of illness (day). Y-axis: each parameter. Abbreviations: FDP: fibrin/fibrinogen degradation products; *, intubation.

**Additional file 4: Supplement Figure S4.** Time course of d-dimer and FDP, d-dimer and fibrinogen level in Case 4. X-axis: day of illness (day). Y-axis: each parameter. Abbreviations: FDP: fibrin/fibrinogen degradation products; *, intubation.

## Data Availability

Data requests should be made to the corresponding authors.

## Abbreviations

COVID-19: Coronavirus disease
SARS-CoV-2: Severe acute respiratory syndrome coronavirus 2
MERS-CoV: Middle East respiratory syndrome
DIC: Disseminated intravascular coagulation
VTE: Venous thromboembolism
PE: Pulmonary embolism
UFH: Unfractionated heparin
LMWH: Low molecular weight heparin
DOACs: Direct oral anticoagulants
APTT: Activated partial thromboplastin time
SIC: Sepsis-induced coagulopathy
ISTH: International Society on Thrombosis and Hemostasis
RT-PCR: Reverse transcriptase–polymerase chain reaction
SLE: Systemic lupus erythematosus
WHO: World Health Organization

## References

[1] Huang C, Wang Y, Li X, Ren L, Zhao J, Hu Y (2020). Clinical features of patients infected with 2019 novel coronavirus in Wuhan, China. Lancet.

[2] Novel Coronavirus (2019-nCoV) situation reports (n.d.) [Available from: https://www.who.int/emergencies/diseases/novel-coronavirus-2019/situation-reports. Accessed 21 Feb 2021.

[3] Iba T, Levy JH, Levi M, Connors JM, Thachil J (2020). Coagulopathy of coronavirus disease 2019. Crit Care Med.

[4] Levi M, Thachil J, Iba T, Levy JH (2020). Coagulation abnormalities and thrombosis in patients with COVID-19. Lancet Haematol.

[5] Cui S, Chen S, Li X, Liu S, Wang F (2020). Prevalence of venous thromboembolism in patients with severe novel coronavirus pneumonia. J Thromb Haemost.

[6] Giannis D, Ziogas IA, Gianni P (2020). Coagulation disorders in coronavirus infected patients: COVID-19, SARS-CoV-1, MERS-CoV and lessons from the past. J Clin Virol.

[7] Klok FA, Kruip M, van der Meer NJM, Arbous MS, Gommers D, Kant KM (2020). Incidence of thrombotic complications in critically ill ICU patients with COVID-19. Thromb Res.

[8] Obi AT, Tignanelli CJ, Jacobs BN, Arya S, Park PK, Wakefield TW (2019). Empirical systemic anticoagulation is associated with decreased venous thromboembolism in critically ill influenza a H1N1 acute respiratory distress syndrome patients. J Vasc Surg Venous Lymphat Disord.

[9] Driggin E, Madhavan MV, Bikdeli B, Chuich T, Laracy J, Biondi-Zoccai G (2020). Cardiovascular considerations for patients, health care workers, and health systems during the COVID-19 pandemic. J Am Coll Cardiol.

[10] Spyropoulos AC, Raskob GE (2017). New paradigms in venous thromboprophylaxis of medically ill patients. Thromb Haemost.

[11] Bartlett RH, Ogino MT, Brodie D, McMullan DM, Lorusso R, MacLaren G (2020). Initial ELSO guidance document: ECMO for COVID-19 patients with severe cardiopulmonary failure. ASAIO J.

[12] Arachchillage DRJ, Passariello M, Laffan M, Aw TC, Owen L, Banya W (2018). Intracranial hemorrhage and early mortality in patients receiving extracorporeal membrane oxygenation for severe respiratory failure. Semin Thromb Hemost.

[13] Raskob GE, Angchaisuksiri P, Blanco AN, Buller H, Gallus A, Hunt BJ (2014). Thrombosis: a major contributor to global disease burden. Arterioscler Thromb Vasc Biol.

[14] Liew NC, Alemany GV, Angchaisuksiri P, Bang SM, Choi G, DA DES, et al. (2017). Asian venous thromboembolism guidelines: updated recommendations for the prevention of venous thromboembolism. Int Angiol.

[15] Asakura H, Ogawa H (2020). Perspective on fibrinolytic therapy in COVID-19: the potential of inhalation therapy against suppressed-fibrinolytic-type DIC. J Intensive Care.

[16] Tang N, Li D, Wang X, Sun Z (2020). Abnormal coagulation parameters are associated with poor prognosis in patients with novel coronavirus pneumonia. J Thromb Haemost.

[17] Zhou F, Yu T, Du R, Fan G, Liu Y, Liu Z (2020). Clinical course and risk factors for mortality of adult inpatients with COVID-19 in Wuhan, China: a retrospective cohort study. Lancet..

[18] Marietta M, Coluccio V, Luppi M (2020). COVID-19, coagulopathy and venous thromboembolism: more questions than answers. Intern Emerg Med.

[19] Lodigiani C, Iapichino G, Carenzo L, Cecconi M, Ferrazzi P, Sebastian T (2020). Venous and arterial thromboembolic complications in COVID-19 patients admitted to an academic hospital in Milan, Italy. Thromb Res.

[20] Martinelli I, Bucciarelli P, Mannucci PM (2010). Thrombotic risk factors: basic pathophysiology. Crit Care Med.

[21] Connors JM, Levy JH (2020). COVID-19 and its implications for thrombosis and anticoagulation. Blood..

[22] McGonagle D, O'Donnell JS, Sharif K, Emery P, Bridgewood C (2020). Immune mechanisms of pulmonary intravascular coagulopathy in COVID-19 pneumonia. Lancet Rheumatol.

[23] Fox SE, Akmatbekov A, Harbert JL, Li G, Quincy Brown J, Vander Heide RS (2020). Pulmonary and cardiac pathology in African American patients with COVID-19: an autopsy series from New Orleans. Lancet Respir Med.

[24] Carsana L, Sonzogni A, Nasr A, Rossi RS, Pellegrinelli A, Zerbi P (2020). Pulmonary post-mortem findings in a series of COVID-19 cases from northern Italy: a two-Centre descriptive study. Lancet Infect Dis.

[25] Wichmann D, Sperhake JP, Lutgehetmann M, Steurer S, Edler C, Heinemann A (2020). Autopsy findings and venous thromboembolism in patients with COVID-19: a prospective cohort study. Ann Intern Med.

[26] Thachil J, Tang N, Gando S, Falanga A, Cattaneo M, Levi M (2020). ISTH interim guidance on recognition and management of coagulopathy in COVID-19. J Thromb Haemost.

[27] Organization WH. (n.d.) COVID-19 Clinical management: living guidance.85. Accessed 21 Feb 2021.

[28] Tang N, Bai H, Chen X, Gong J, Li D, Sun Z (2020). Anticoagulant treatment is associated with decreased mortality in severe coronavirus disease 2019 patients with coagulopathy. J Thromb Haemost.

[29] Chan AK, Paredes N, Thong B, Chindemi P, Paes B, Berry LR (2004). Binding of heparin to plasma proteins and endothelial surfaces is inhibited by covalent linkage to antithrombin. Thromb Haemost.

