# Supplementary figures and images for: An evaluation of venous thromboembolism by whole-body enhanced CT scan for critical COVID-19 pneumonia with markedly rises of coagulopathy related factors: a case series study
Source: Thromb J. 2021 Apr 20;19:26. doi: 10.1186/s12959-021-00280-z (PMC8057658; doi:10.1186/s12959-021-00280-z)

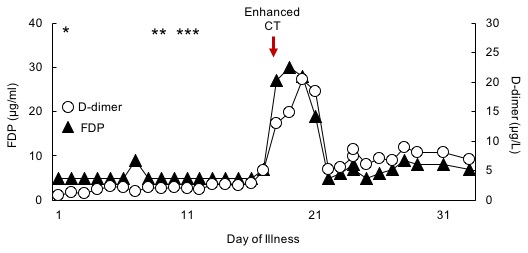

Supplement: Supplementary file 1 — Additional file 1: Supplement Figure S1. Time course of d-dimer and FDP level in Case 1. X-axis: day of illness (day). Y-axis: each parameter. Abbreviations: FDP: fibrin/fibrinogen degradation products; *, intubation, **, extubation, ***, discharge from intensive care unit. [file 12959_2021_280_MOESM1_ESM.jpg]

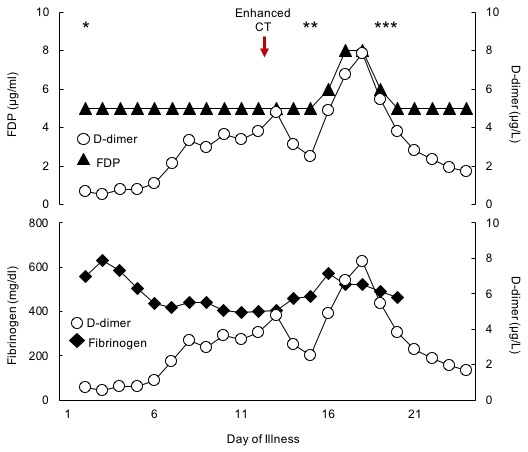

Supplement: Supplementary file 2 — Additional file 2: Supplement Figure S2. Time course of d-dimer and FDP, d-dimer and fibrinogen level in Case 2. X-axis: day of illness (day). Y-axis: each parameter. Abbreviations: FDP: fibrin/fibrinogen degradation products; *, intubation, **, extubation, ***, discharge from intensive care unit. [file 12959_2021_280_MOESM2_ESM.jpg]

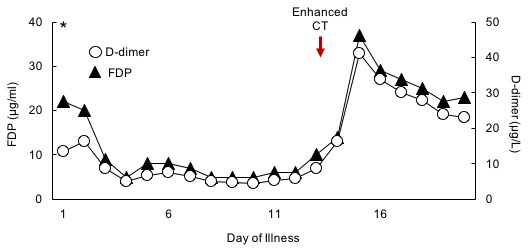

Supplement: Supplementary file 3 — Additional file 3: Supplement Figure S3. Time course of d-dimer and FDP level in Case 3. X-axis: day of illness (day). Y-axis: each parameter. Abbreviations: FDP: fibrin/fibrinogen degradation products; *, intubation. [file 12959_2021_280_MOESM3_ESM.jpg]

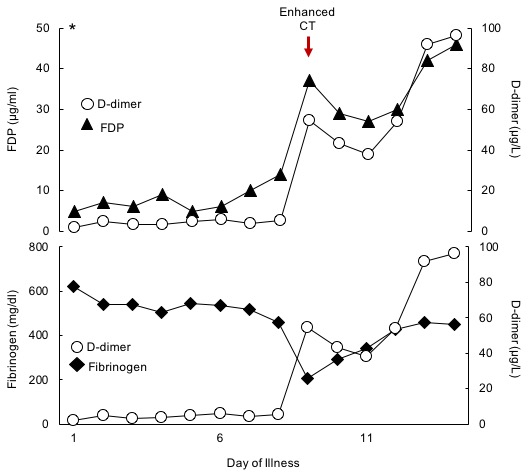

Supplement: Supplementary file 4 — Additional file 4: Supplement Figure S4. Time course of d-dimer and FDP, d-dimer and fibrinogen level in Case 4. X-axis: day of illness (day). Y-axis: each parameter. Abbreviations: FDP: fibrin/fibrinogen degradation products; *, intubation. [file 12959_2021_280_MOESM4_ESM.jpg]
